# Research and evaluation on students’ emotional attachment to campus landscape renewal coupling emotional attachment scale and public sentiment analysis: a case study of the “Heart of Forest” in Beijing Forestry University

**DOI:** 10.3389/fpsyg.2023.1250441

**Published:** 2023-09-25

**Authors:** Ruoshi Zhang

**Affiliations:** School of Landscape Architecture, Beijing Forestry University, Beijing, China

**Keywords:** emotional attachment, campus landscape renewal, emotional attachment scale, public sentiment analysis, Heart of Forest

## Abstract

In the era of stock renewal, the construction of university campuses in China’s first-tier cities has shifted from demolition and construction to renewal and upgrading, in which public landscape space is the main environment for students’ daily life, learning and entertainment. Especially during the outbreak of the recent COVID-19 epidemic, it has become an important way for students to interact with nature and obtain emotional healing. In the existing studies, there is a lack of discussion on the correlation between the spatial characteristics of the updated campus landscape and students’ emotional attachment, and there are few quantitative studies. Based on this, this paper takes the “Heart of Forest” landscape space as an example, and integrates multi-dimensional quantitative methods including emotional attachment scale and public semantic analysis to study and evaluate the characteristics of landscape space that affect students’ emotional attachment. The results show that: (1) Overall, the landscape space renewal of the Heart of Forest provides students with positive emotional experiences and effectively enhances students’ emotional attachment as well as sense of belonging to the campus. (2) Among them, the material characteristics of the site including nature-related elements, materials, structures play a positive role in promoting the vast majority of students in the process of establishing emotional attachment, which is particularly obvious for students majoring in landscape, architecture and urban planning. (3) Whether the public social space can effectively provide students with a good emotional experience is closely related to the frequency and purpose of students’ use of the space. (4) The interactive characteristics such as changeability and playability fail to promote emotional attachment because of lacking of management and maintenance. The renewal and transformation of the “Heart of Forest” landscape space is generally successful in promoting students’ emotional attachment, and provides a reference for the future campus landscape renewal design from different angles. In addition, the quantitative study of emotional attachment constructed in this paper coupled with multi-dimensional data provides a method for the evaluation of students’ emotional experience of campus landscape.

## 1. Introduction

In recent years, China’s urban development has entered a stage of stock renewal (Yi et al., 2017; Ye et al., 2021). Under this circumstances, in the core areas of first-tier cities such as Beijing, where land is tight, the main way public education spaces development with large areas especially university campuses has also shifted from new construction and reconstruction to renewal and upgrading (Guo W. et al., 2022; Yuanshuo, 2023). Among them, the renewal of public landscape space on campus as the main place for students’ extracurricular activities, rest and socialization is closely related to the physical and mental health of college students, and the design of good natural landscape and cultural landscape has also been proved to enhance students’ emotional attachment and satisfaction with the campus environment (Lau and Yang, 2009; Andre et al., 2017; Vella-Brodrick and Gilowska, 2022). During the outbreak of COVID-19 in the past 3 years, the campus landscape space has especially become an important psychological healing space and emotional anchor point for students whose life, recreation and learning activities are confined to the campus space (Biswas and Sen, 2020; Lai et al., 2020; Rousseau and Deschacht, 2020; Liu et al., 2022). Therefore, the research and evaluation of the landscape space transformation on campus from the perspective of emotional attachment are of great value to both the improvement of students’ wellbeing and the future campus construction in the context of stock renewal in China.

In the existing research on the relationship between landscape transformation and emotion, most of the researches focus on urban scale public spaces, and the types and ages of the research population are widely distributed, and few focuses on landscape renewal and young student groups on campus (Kara, 2013; Kong et al., 2022; Zeng et al., 2022; Yan et al., 2023). Relating researches mainly focuses on landscape design methods, and the discussion of emotions is mostly reflected in qualitative descriptions, lacking objective quantitative evaluation. This may be also due to the complexity and diversity of emotions. At this time, the multidisciplinary emotional attachment scale method and semantic analysis method of social media data with help of big data technologies provide opportunities to improve the quantitative research of emotional attachment (Zhang, 2023).

Based on this, the study takes the “Heart of the Forest” landscape space in Beijing Forestry University as an example. The space was renovated and put into use in 2020. The study couples the top-down targeted research using emotional attachment scale and the bottom-up emotional semantic analysis using social media big data, to quantify the emotional attachment of students to different landscape features and spatial elements in the landscape renewal and transformation of universities. On the one hand, the research complements the lack of quantitative research methods for the emotional attachment between college students and landscape space, and on the other hand, it also provides a more scientific and objective reference for the campus landscape renewal design that cares for students’ emotional experience in the era of stock renewal.

## 2. Literature review

### 2.1. The effect of natural landscape to students’ emotional attachment on campus

The value of natural landscape for human well-being and sustainability has been widely studied and recognized. Back to 1995, the Biophlia Hypothesis proposed by Wilson had assumed that human had innate need for natural environment as they born and evolved with it (Kellert and Wilson, 1995). Later, psychological evolutionary theories including Stress Reduction Theory (SRT) and Attention Restoration Theory (ART) have been developed based on this hypothesis, the former suggests that visiting and contacting with nature helps shift stress states to positive emotional states while discourage negative thoughts, and the later suggests that natural environment help improve cognitive performance and restore attention (Kaplan and Kaplan, 1989; Kaplan, 1995; Jiang et al., 2021). Steven Kaplan and Rachel Kaplan further emphasizes that unlike urban environment, nature is filled with more intriguing stimuli which may grabs attention in a bottom-up fashion. Its ability to establish emotional connection with people can be analyzed in terms of its evolutionary significance. Based on this, the importance of human beings themselves has been highlighted (Kaplan et al., 1998), and the relevant research on the impact of nature on people has begun to further deepen into people’s multi-dimensional perception of nature, such as through viewing, smelling, touching, hearing, tasting, etc., people can get different degrees of emotional healing from the natural environment. For example, merely viewing plants or even pictures of nature has been proved to promote people’s positive emotion (Lee et al., 2009; Honold et al., 2016). Research from neuroscience also proves this (Van den Berg, 2008). Besides, interacting with natural plants, touching natural materials and hearing natural sounds has also significantly enhanced people’s positive experience (Koga and Iwasaki, 2013; Ratcliffe, 2021; Rickard and White, 2021). Nowadays, the supply of natural landscape such as urban greenspaces plays an important role in daily public life, as they serve as a place for exercise, relaxing, as well as natural exposures (McPherson, 1992; Van Leeuwen et al., 2010; Zhu et al., 2021). During COVID-19 pandemic especially city lockdowns, they have become the main space for both people’s physical and psychological healing, and their recreational usage is increasing due to social limitations brought by such public health crisis. Multiple researches have reported that greenspace in cities such as parks provided positive contributions to self-reported well-being during the COVID-19 pandemic (Xie et al., 2020; Guo X. et al., 2022). Therefore, greenspace design and construction has become an important humanized renewal method in the current era of stock renewal.

However, few have delved specifically into the interaction between the greenspaces on university campuses and students’ physical and mental health. It has been proved by multidisciplinary researchers that a positive university environment creates conditions for students’ effective learning, academic achievements, fewer emotional problems and promotes students’ overall wellbeing, of which the interaction with the environment itself plays an important role (Brandisauskiene et al., 2021; Vella-Brodrick and Gilowska, 2022). This is particularly prominent in research on students’ emotional demands on the university environment during the COVID-19 pandemic (Liu et al., 2022). Therefore, exploring the relationship between the specific characteristics of campus green space and students’ emotional attachment is conducive to further promoting the design and renewal methods of a sustainable campus environment that truly cares for students’ wellbeing.

### 2.2. The definition and evaluation of emotional attachment between people and landscape

The study of emotional attachment is promoted by multidisciplinary researchers, including those involved in psychology, human geography, architecture, landscape architecture and urban planning (Scannell and Gifford, 2010; Lewicka, 2011; Ekkekakis, 2013; Adams, 2015; Scannell and Gifford, 2017; Liu et al., 2020; Birnbaum et al., 2021; Arnberger et al., 2022; Zhang et al., 2022). It discusses the relationship between people and certain environment based on emotion, cognition, and behavior. Emotions are the primary focus (Altman and Low, 2012; Adams, 2015). For different research objects, researchers have constructed multi-angle and multi-dimensional theoretical frameworks. Among them, the place attachment theory (PA) systematically and completely discusses the influence factors, as well as indicators of the emotional attachment between people and specific environment characteristics (Lewicka, 2011). Based on this, researchers in different fields have carried out different explorations. In the field of psychology and human geography, researchers explore the mechanism of the establishment of emotional attachment and construct multi-dimensional attachment measurement scales, of which different indicators promoting emotional attachment are emphasized (Williams and Roggenbuck, 1989; Altman and Low, 2012; Scannell and Gifford, 2013; Adams, 2015). Meanwhile, researchers from architecture, urban planning, landscape architecture and other fields related to environmental studies and design pay more attention to the different roles of the built environment and the natural environment on emotional attachment. For example, the place-based phenomenological research and the exploration of the meaning of people-built environment interactions are conducted to study people’s sense of identity with buildings or cities (Tuan, 1979; Carmona, 2019; Feng et al., 2021; Arnberger et al., 2022). As for natural environment, the above mentioned Stress Reduction Theory (SRT) and Attention Restoration Theory (ART) have started to raise the importance of nature to emotion. Subsequently, some scholars construct models to analyze the relationship between place attachment and the natural environment, such as the intrinsic relationship between environmental preference, place attachment and restorative evaluation, and the resilience of the natural environment with a high degree of place attachment is higher than that of the urban environment (Lin et al., 2019; Liu et al., 2020; Birnbaum et al., 2021; Feng et al., 2021; Zhang, 2021; Arnberger et al., 2022). Whether in China or abroad, most of their research subjects are concentrated in urban renewal and landscape renewal. This is precisely because, in the context of stock renewal, the response of place attachment to the contradiction between people and land, the preservation of the context of the place and the promotion of public participation are of great value. This also supports the value of this theory for this study.

The method of using the scale to measure people’s emotion is derived from the emotional attachment theories represented by Place Attachment Theory, and the scales used in different researches are constructed based on different research objects, problems or goals. Based on existing multidisciplinary researches, the measurement of attachment to landscape mainly includes the following three aspects: the dimension of emotional attachment between people and the landscape (positive or negative), the degree of emotional attachment, and the emotional attachment to specific landscape characteristics. They also correspond to the different goals of the researchers under different background.

The first two goals could be achieved with the help of the Positive and Negative Analysis Scale (PANAS) and the Place Attachment Scale. The positive and negative analysis scale (PANAS) is widely used to explore whether the strength of attachment to a certain space is closely related to the positive and negative emotions. It consists of 10 positive and 10 negative emotional adjectives, and is measured by Likert 5-point scoring method, of which 1 point indicates that there is almost no such emotional experience, and 5 points indicates that such emotional experience is strong. For the latter, the person-process-place (PPP) framework of place attachment measuring scale has shown high reliability and validity and has been used by scholars in multi-scale environment as well as landscape researches in the past 2 years. The Place Attachment Scale consists of 20 items, based on Likert’s 7-point scoring method, of which 1 point indicates the least disagreement with the narrative content of the question and 7 points indicates strong agreement with the narrative content of the question.

Meanwhile, in order to explore the emotional influence of specific characteristics of the targeted landscape, researchers often construct scales based on research needs. In the field of built environment design and research, semi-structured measurement scales are formed based on the pre-study of the specific landscape characteristics. The scales mainly include the physical characteristics, social characteristics and interactive characteristics. The specific factors for each part vary slightly from study to study depending on the object. The Likert scale is also used to quantitatively measure the specific intensity of each characteristic, with a score of 1 indicating almost no emotional attachment to this characteristic and a score of 7 indicating a strong emotional attachment.

### 2.3. The application of semantic analysis in the evaluation of emotional attachment between people and landscape

Although the above emotional attachment scale provides a means to quantify the dimension and degree of emotion, the amount of data obtained is still limited, and its top-down construction logic cannot completely avoid the researcher effect. In the era of information and communication technology, visualizing sentiment based on social media data (SMD) provides a bottom-up quantitative research approach for evaluating human emotions, which is an effective supplement to the top-down scale method (Chakraborty et al., 2020; Jindal and Aron, 2021). SMD serve as a reliable and relatively objective data source to reflect the public’s attitudes toward a certain landscape (Barbier and Liu, 2011; Sim et al., 2020). Among them, college students are the most active group of people who use these social platforms to express their feelings. This provides a data basis for the intervention of semantic analysis in this study.

The development and universalization of social media has changed the way people interact with environment, making it useful for exploring public preference for a landscape or place (Wilkins et al., 2020; You et al., 2022). Based on this, sentiment analysis is used to qualify and classify people’s emotions during their interaction with landscape, and then define different degree of sentiment values as different emotions, which helps reveal the benefits of landscape. For example, Twitter sentiment and georeferenced Flickr tweets have been used in New York and Sheffield, respectively, to measure well-being brought by urban park and the quality of urban greenspaces. The results have shown that high sentiment in landscape relates to high positive emotions, which implies higher satisfaction (Brindley et al., 2019). Meanwhile, SMD from the two most popular social networking platform in China, Tecent and Sina Weibo, have been used to explore both people’s emotional features and preference in different kinds of landscapes, such as emotional enthusiasm enhanced by greenspaces (Guo X. et al., 2022) and positive emotions brought by forest landscape (Zeng et al., 2022). Although the existing research fully supports the effectiveness of semantic analysis in evaluating the emotional attachment characteristics between people and landscapes, few studies have paid special attention to the emotional appeal and evaluation of campus landscape by college students. It is vital to explore and understand these students’ emotional demands to improve campus landscape to match needs, especially under the background of stock renewal.

## 3. Materials and methods

### 3.1. Study area

**Figure 1:**
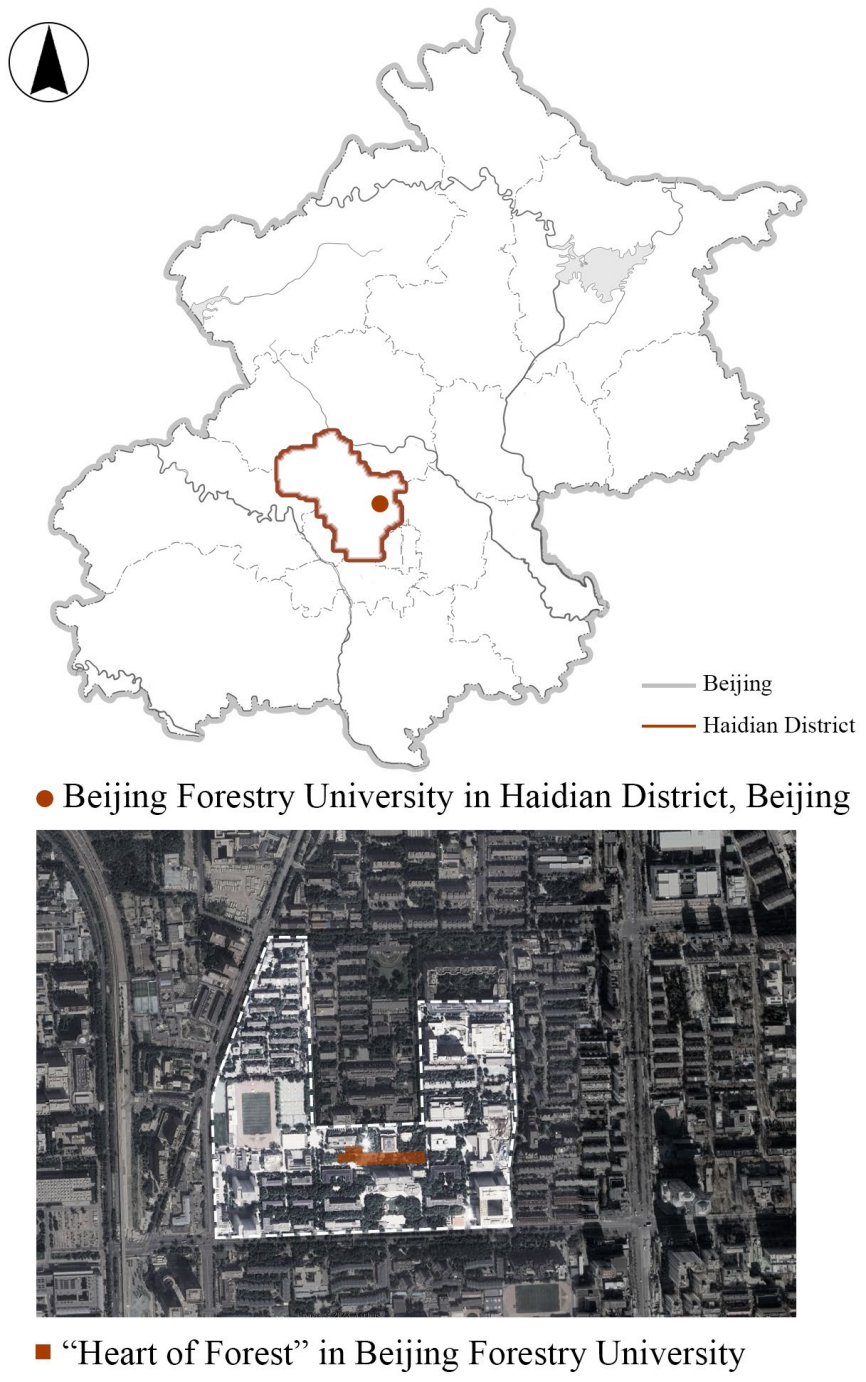
The location and area of the Heart of Forest.

**Figure 2:**
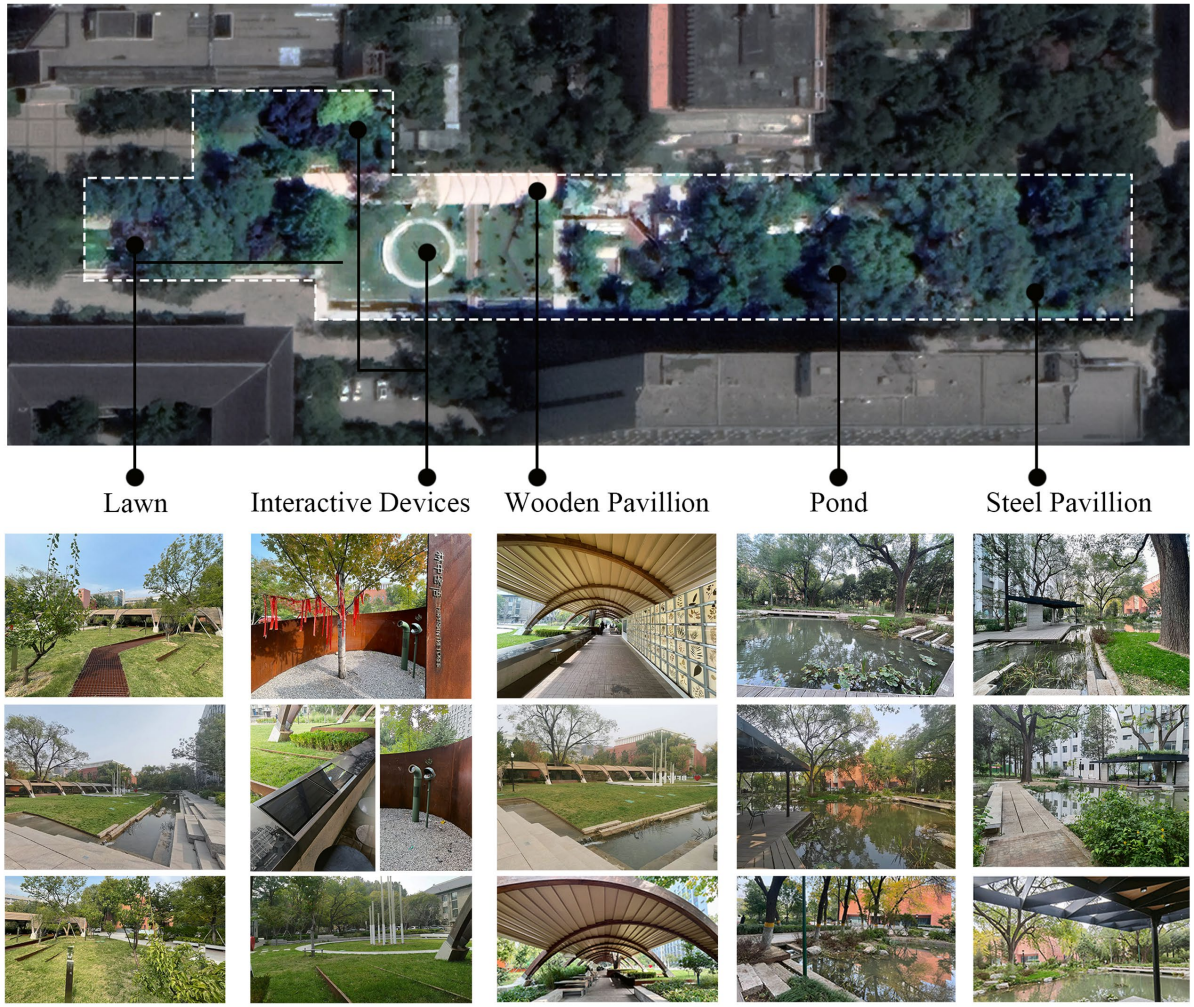
The representative features of each part in the Heart of Forest (photos taken by author).

The study area, “Heart of Forest,” is located in the center of Beijing Forestry University, covering a total area of about 12,000 square meters (Figure 1). This campus landscape space is the largest public activity space in Beijing Forestry University, which was completed and put into use in September 2020. Heart of Forest is a landscape renewal project after the demolition of the temporary building of the original school hospital, its transformation and design consists of three aspects: natural land-scape, artificial landscape, and interactive landscape. The natural landscapes include ponds, rainwater harvesting devices, lawns, old elm trees, and garden plants. The cultural and artificial landscape includes pavilions, seats, and a commemorative installation of the former school hospital. The interactive landscape includes herbarium walls, interactive light pillars, and voice recording devices. These landscape spaces promote students’ emotional attachment to the Heart of Forest by providing them with a variety of emotional experiences through natural, cultural, historical, and behavioral inter-actions. Since its completion, the Heart of Forest has become the most popular place among students for leisure, activities and communication on the campus of Beijing Forestry University, which makes it a perfect area for this study. Combined with its design concept and landscape composition elements, the landscape characteristics that trigger emotional attachment can be integrated into three categories: material, cultural and interactive, including: material, color, natural elements, form and structure, privacy, diversity, sociability, territoriality, playability, uniqueness, and changeability. This also provides a basis for the construction of the emotional attachment scale of specific landscape composition characteristics in subsequent research. The specific features of each part of the Heart of Forest can be seen in Figure 2.

### 3.2. Data sources

#### 3.2.1. Emotional attachment scale data

The emotional attachment scale was randomly distributed to students who visited the Heart of Forest to measure the degree and dimension of students’ emotion when they were there. The data collection began in October 2022 and lasted about 2 weeks. The time period was under the epidemic of COVID 19 then, in order to ensure safety, almost all the students returning to school studied and lived on campus, and the Heart of Forest became the main public space for students to rest and communicate after class, alleviating the anxiety caused by the epidemic, which especially highlighted the emotional healing value of the Heart of Forest. A total of 159 scales were collected, of which 143 were with valid data. Since the completion of each scale required the semi-structured guidance of a researcher, it took participants about 20 min to complete the scale, and campus activities were seriously limited during the epidemic of COVID-19 then, the scale of available valid data was the maximum amount that can be obtained with limited time and limited manpower. According to previous research, men and women in general have different characteristics of emotional attachment to the same landscape. Meanwhile, as the subject of this study, the Heart of Forest, is located inside the Beijing Forestry University, and its designer is a teacher of the School of Landscape Architecture, so students studying landscape, architecture and urban planning may have different emotional attachment when interacting with landscape. At the same time, whether or not one purposefully interacts with the environment has also been shown to affect the degree of emotional attachment to the environment. Based on this, the study also collected information of participants’ major and purpose of visit. The gender, major distribution and whether this is a purposeful visit of the participants can be found in the table. Valid data is entered into IBM SPSS V25.0 (International Business Machines Corporation (IBM), IBM headquarters in Almonk, New York, NY, USA) for subsequent analysis.

#### 3.2.2. Social media data

The text data associated with the Heart of Forest from Sina Weibo and Little Red Book (XiaoHongShu) was used for emotional semantic analysis in this study. Sina Weibo (Beijing, China) is the most popular platform for people to distribute and access information, especially college students. According to 2020 Weibo User Development Report, Weibo’s monthly active users and daily active users reached 511 million and 224 million respectively, of which the post-90s and post-00s accounted for more than 80%, which makes it one of the most visited websites in mainland China as well as a perfect platform for accessing Weibo post data of students (Sina Weibo, 2020). Weibo post data refers to text information posted by Weibo users at a given place, which in this research is the Heart of Forest in Beijing Forestry University. The data is used to quantify students’ emotions when they take series activities in the Heart of Forest. These data were obtained from the Sina Weibo application programming interface (API)^1^ with the help of Python (version 3.7). The data cover the time range from September 1, 2020 when the Heart of Forest was open to students, to October 30, 2022. Same as Weibo, Little Red Book is a lifestyle sharing platform, which also allows users to share short videos and photos about fashion, beauty, food, travel, and much more. According to Qiangua’s 2022 XiaoHongshu Active User Portrait Trend Report (2022), the number of monthly active users of Little Red Book reached 200 million in 2022, with 72% of users being born in the 1990s. The data obtained from Little Red Book include log and comment text relating to the Heart of Forest in Beijing Forestry University, which were collected from the API of https://www.xiaohongshu.com/ using Python programming language. The data cover the same time range of Weibo post data above (from September 1, 2020 to October 30, 2022).

The original Weibo and Little Red Book text contains various redundant information, including punctuation marks, subject tags, hyperlinks, and @other-users tags in Weibo. Python was used in data cleaning process to eliminate errors while improve the efficiency of word sentiment in sentiment analysis. The cleaned data were visualized using SPSS, and the location tagged with the Heart of Forest of Beijing Forestry University was selected to obtain 3,702 Weibo and Little Red Book data.

### 3.3. Build a methodology

#### 3.3.1. Emotional attachment scale design and analysis

Based on the aforementioned review of the application of the emotional attachment scale, firstly, the purpose of this study should be clarified. There are three goals to quantitatively evaluate the emotional attachment between students and landscape of the Heart of Forest: the dimension of emotional attachment between students and the Heart of forest (positive or negative), the degree of emotional attachment, and the emotional attachment to specific characteristics consisting of the Heart of Forest.

Therefore, the Positive and Negative Analysis Scale (PANAS) and the Place Attachment Scale were chosen to achieve the first and second goals, and a newly constructed scale aiming at detailed characteristics of the Heart of Forest were designed to achieve the third goal. The former two scales met the aim of this study both in content and logic by emphasizing the importance of the environment itself in the attachment process. In order to avoid a cultural gap and improve the clarity of the scale, a Chinese version of the two scales was revised. Meanwhile, in order to explore the emotional influence of specific characteristics of the landscape of the Heart of Forest, a semi-structured measurement scale was formed based on the pre-study of landscape characteristics in the Heart of Forest. As a result, the physical characteristics including material, color, natural plants, form and structure, and social characteristics including privacy, diversity, sociability, territoriality, and interactive characteristics that including playability, uniqueness, and changeability were chosen as indicators.

IBM SPSS V25.0 (International Business Machines Corporation (IBM), IBM headquarters in Almonk, New York, NY, USA) was used for data processing after the data collection using the above mentioned scales. The analysis consisted of three steps.

The first step was the description of basic characteristics of the data representing comprehensive degree and intensity of attachment. Arithmetic average was used to describe the extent of positive ( $A_{L}$ ) and negative emotion ( $A_{N}$ ), place attachment ( $A_{PA}$ ), and overall attachment to landscape characteristics of the Heart of Forest ( $A_{LC}$ ). Standard deviation was used to describe the magnitude of the difference in participants’ positive ( $S_{P}$ ) and negative emotion ( $S_{N}$ ), place attachment ( $S_{PA}$ ), and attachment to overall landscape characteristics of the Heart of Forest ( $S_{LC}$ ). The formula was as follows:

(1) $$A_{\left(P,N,PA,LC\right)}=\frac{1}{n}\overset{n}{\underset{i=1}{\sum }}b_{{\left(P,N,PA,LC\right)}_{i}}$$

(2) $$S_{\left(P,N,PA,LC\right)}=\sqrt{\frac{\overset{n}{\underset{i=1}{\sum }}{\left(b_{{\left(P,N,PA,LC\right)}_{i}}-\bar{b_{\left(P,N,PA,LC\right)}}\right)}^{2}}{n-1}}$$

where $b_{\left(P,N,PA,LC\right)i}$ represents the score of the i th participating student on each item in the four components of the above emotional attachment scale: positive affect ( $b_{P}$ ), negative affect ( $b_{N}$ ), place attachment ( $b_{PA}$ ), and attachment to overall landscape characteristics ( $b_{LC}$ ). Among them, $b_{P}$ is the arithmetic average of the scores scored by the 10 words on the PANAS scale that describe positive emotions, $b_{N}$ is the arithmetic average of the scores scored by the 10 words on the PANAS scale that describe negative emotions, $b_{PA}$ is the arithmetic mean of the scores scored by all items on the PA scale, and $b_{LC}$ is the arithmetic mean of the scores scored by each specific landscape characteristic on the attachment intensity to landscape characteristics scale. n is 144 in this study.

Additionally, the correlation between positive and negative affect, place attachment, and specific landscape characteristics was explored using correlation analysis (Pearson) based on SPSS. The assessment of the normality of data is conducted before correlation analysis using SPSS. The above arithmetic average result of positive affect, negative affect, place attachment and scores of the intensity of attachment to each landscape characteristic of each participant are used here as specific value to calculate the Pearson correlation coefficient r :

(3) $$r=\frac{n\left(\sum xy\right)-\left(\sum x\right)\left(\sum y\right)}{\sqrt{\left[n\sum x^{2}-{\left(\sum x\right)}^{2}\right]\left[n\sum y^{2}-{\left(\sum y\right)}^{2}\right]}}$$

where the variables to both x and y are the same, including arithmetic average result of positive affect, negative affect, place attachment and scores of the intensity of attachment to each landscape characteristic of each participant.

Last but not least, with the help of exploratory factor analysis (EFA) in SPSS, the spatial characteristics contributing to attachment of the Heart of Forest were summarized into systematic spatial dimensions. Exploratory factor analysis (EFA) is used to identify complex interrelationships among items and group items that are part of unified concepts. There’s no “*a priori*” assumptions have been made about the relationships among the emotional attachment factors. Extraction method in the analysis is Principal Component Analysis (PCA) with Varimax rotation method.

#### 3.3.2. Sentiment analysis based on sentiment dictionaries

**Figure 3:**
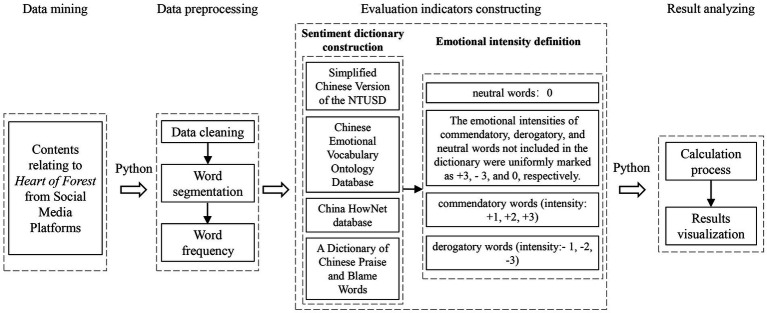
Sentiment analysis process.

Identifying students’ opinions and preference on school landscape transformation plays an important role in updating the existing campus environment and planning the future campus of Beijing Forestry University under the background of stock renewal. In this study, the textual content of Weibo and Little Red Book was used to analyze the sentiments of students who had experiences in the landscape of Heart of Forest from bottom up to avoid the researcher effect as well as provide support for the scale analysis above. Text sentiment analysis is a widely used tool for extracting subjective information from natural language texts and classify them into sentiment categories, such as positive, negative and neutral. The two most commonly used sentiment methods in text sentiment analysis are analysis using sentiment dictionaries and machine learning. According to the feature of the texts in this study, sentiment analysis methods based on sentiment dictionaries were chosen to collect and analyze students’ attitudes and sentimental responses to different landscape characteristics of the Heart of Forest. Specifically, frequency in text messages reflects the attention of students to relevant landscape characteristics, and the size of emotional computing data reflects the intensity of students’ emotional experience. The process of sentiment semantic analysis is shown in Figure 3.

Texts about the Heart of Forest were selected from the above mentioned two public social media platforms: Weibo and Little Red Book. Python was used to perform data mining and cleaning of the collected texts. A total of 3,702 valid sample data points were obtained. The NLPIR-Parser big data semantic intelligent analysis platform was used to preliminarily segment the comment content, and after filtering out meaningless words, statistical results for high-frequency words were obtained, and high-frequency words with the same semantics were merged.

High-frequency words classified using different evaluation scales and A Dictionary of Chinese Praise and Blame Words were screened by intersection with the Simplified Chinese Version of the National Taiwan University Sentiment Dictionary, the Chinese Emotional Vocabulary Ontology Database, and the China HowNet database (CNKI). Then, similar words were merged using A New Chinese Synonym Forest. After screening, the intensity of each word was used to define its emotional intensity, and the evaluation indicators were divided into four types: commendatory words (intensity: +1, +2, +3), derogatory words (intensity: −1, −2, −3), neutral words (intensity: 0), and special words (words not included in the dictionary). The emotional intensities of commendatory, derogatory, and neutral words not included in the dictionary were uniformly marked as +3, − 3, and 0, respectively.

#### 3.3.3. Methodology framework

**Figure 4:**
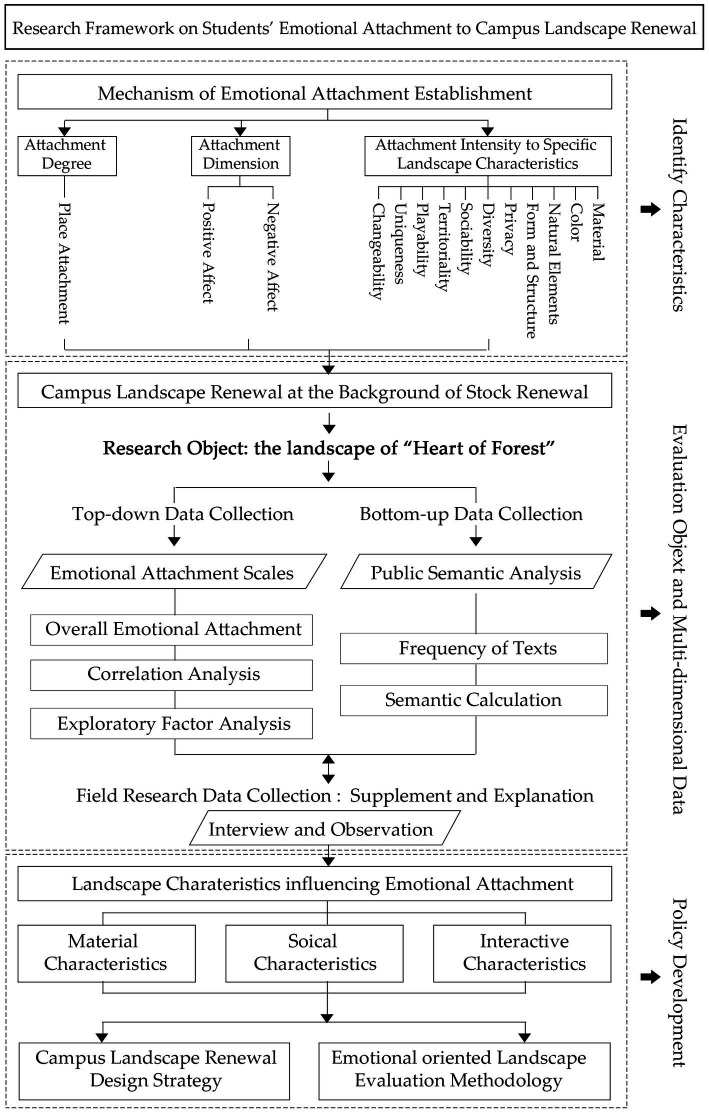
The framework of the study.

Figure 4 illustrate the framework of this study. The study focuses on the research and evaluation on students’ emotional attachment to campus landscape renewal coupling emotional attachment scale and public sentiment analysis. The landscape of Heart of Forest in Beijing Forestry University is chosen as research object. The study adopts the logical path of “criteria establishment-methodology construction-results processing” to evaluate the emotional attachment of the updated landscape space and comprehensively analyze the value of specific landscape characteristics with the results of scale analysis and sentiment analysis.

## 4. Results

### 4.1. The results of emotional attachment scale analysis

#### 4.1.1. The overall emotional attachment status to the Heart of Forest

The data of all participants and data based on gender and major differentiation of emotional attachment to the Heart of Forest is listed in Table 1. As for attachment degree represented by Place Attachment, the results showed that regardless of gender and major, students generally established a relatively strong place attachment to the Heart of Forest, as the average degree exceeded the mean value, while the value of standard deviation of the total data indicated that this attachment degree was quite different between different student. Among them, female students had stronger place attachment than male students, while students majoring in environmental design related majors had significantly stronger attachment to the Heart of Forest than students of other majors. As for attachment dimension including positive affect and negative affect, the positive affect obtained by students was overall more pronounced than the negative affect. Among them, female students have stronger positive emotions and weaker negative emotions than male students. As for students’ attachment intensity to landscape characteristics of the Heart of Forest, the results of mean value showed that they generally have a strong emotional experience of the characteristics of the landscape and environment themselves, which once again proves the emotion induced value of the specific characteristics of this designed landscape. For different genders, male students had a stronger emotional experience of landscape characteristics, while for different majors, students majoring in environmental design related majors continued to show a stronger emotional tendency toward landscape itself.

**Table 1:** The overall emotional attachment status.

Emotional attachment	Total		Male		Female		Environment design related majors	
	Mean	SD	Mean	SD	Mean	SD	Mean	SD
Positive affect	2.88	0.85	2.84	0.91	2.89	0.82	2.93	0.79
Negative affect	1.39	0.59	1.60	0.88	1.31	0.38	1.34	0.46
Place attachment	4.42	0.87	4.39	0.85	4.43	0.88	4.65	0.84
Attachment intensity to landscape characteristic	5.31	0.94	5.35	0.87	5.29	0.98	5.49	0.87

#### 4.1.2. The correlation analysis results

The correlation analysis was processed to further explore the extent to which each specific landscape characteristic contributed to the degree and dimension of emotional attachment. Based on the result of p value for the S-W test and Q-Q plot of the tests of normality, the 14 variables are approximately normally distributed. The correlation coefficients have been calculated among all 14 objects. The results of the overall participants, male, female, and participants from environment design related majors are shown in Tables 2–5. According to Table 2, It was evident that students’ overall attachment to the Heart of Forest was positively correlated with positive affect (0.553**). The results describing both the material and social characteristics of the landscape were significantly positively correlated with place attachment, as they all have a relatively high correlation coefficient at 0.01 (double-tailed). This demonstrated the value of the landscape design and construction of the campus environment renewal with respect to emotional attachment. Comparing the correlation strength between the material characteristics of the Heart of Forest as well as the social and interactive characteristics and emotional attachment, it could be found that the social and interactive characteristics have a greater effect on emotional attachment than the material characteristics, as five of the correlation factors of the former were greater than 0.5, including diversity, sociability, territoriality, playability, and uniqueness (0.587**, 0.561**, 0.537**, 0.651**, 0.560**), while in the latter, only the formal and structural characteristics are greater than 0.5 (0.561**), and the natural element characteristics are about 0.5 (0.498**). This showed that in the campus landscape renewal, providing students with comfortable activity venues and setting up interactive and diverse landscape facilities were conducive to promoting students’ emotional experience. Focusing on the correlation calculation results between the detailed landscape features, it could be found that the form and structure were significantly correlated with the materials, colors and natural elements in the landscape space of the Heart of Forest, meanwhile the materials and colors were significantly correlated with natural elements, which indicated that the use of natural materials and the method of expressing the texture of the material itself during the design and construction of landscape was conducive to the establishment of its emotional association with users, such as the wooden pavilion and the interactive space enclosed by rusty steel plates in the Heart of Forest. Meanwhile, it could be found that diversity and materials was significantly correlated, indicating that the combination of various materials that made up the landscape of the Heart of the Forest, such as wood, rusty steel plate, natural stone, and natural elements such as water, plants, pebbles, etc., effectively promoted the formation of emotional attachment. The significant correlation between the uniqueness of and the material, form and structure of the Heart of Forest further indicated that the pavilions and spatial nodes composed of the latter were important driving forces for the formation of uniqueness, and thus enhance the emotional experience of students.

**Table 2:** Correlation coefficients of total participants.

		1	2	3	4	5	6	7	8	9	10	11	12	13	14
1	Place attachment	1													
2	Positive affect	0.553**	1												
3	Negative affect	−0.023	0.196*	1											
4	Material	0.445**	0.266**	−0.197*	1										
5	Color	0.459**	0.241**	−0.137	0.678**	1									
6	Natural elements	0.498**	0.253**	−0.252**	0.678**	0.646**	1								
7	Form and structure	0.561**	0.291**	−0.188*	0.642**	0.682**	0.685**	1							
8	Privacy	0.342**	0.224**	−0.058	0.362**	0.295**	0.242**	0.353**	1						
9	Diversity	0.587**	0.414**	−0.139	0.617**	0.515**	0.570**	0.578**	0.443**	1					
10	Sociability	0.561**	0.371**	−0.007	0.340**	0.334**	0.467**	0.497**	0.260**	0.565**	1				
11	Territoriality	0.537**	0.257**	−0.198*	0.502**	0.320**	0.383**	0.407**	0.332**	0.549**	0.416**	1			
12	Playability	0.651**	0.402**	−0.046	0.404**	0.261**	0.349**	0.406**	0.286**	0.570**	0.509**	0.457**	1		
13	Uniqueness	0.560**	0.393**	−0.183*	0.633**	0.565**	0.619**	0.692**	0.371**	0.663**	0.521**	0.529**	0.590**	1	
14	Changeability	0.416**	0.256**	−0.046	0.454**	0.380**	0.384**	0.484**	0.446**	0.512**	0.328**	0.392**	0.445**	0.560**	1

*p < 0.05 (2-tailed), **p < 0.01 (2-tailed).

**Table 3:** Correlation coefficients of male participants.

		1	2	3	4	5	6	7	8	9	10	11	12	13	14
1	Place attachment	1													
2	Positive affect	0.525**	1												
3	Negative affect	−0.087	0.120	1											
4	Material	0.304	−0.026	−0.324*	1										
5	Color	0.368*	0.226	−0.160	0.614**	1									
6	Natural elements	0.459**	0.075	−0.430**	0.566**	0.556**	1								
7	Form and structure	0.450**	0.131	−0.503**	0.452**	0.506**	0.632**	1							
8	Privacy	0.129	0.150	−0.052	0.181	0.167	0.053	0.297	1						
9	Diversity	0.377*	0.233	−0.249	0.471**	0.441**	0.465**	0.424**	0.339*	1					
10	Sociability	0.536**	0.234	−0.255	0.329*	0.452**	0.611**	0.637**	0.212	0.643**	1				
11	Territoriality	0.467**	0.080	−0.286	0.420**	0.119	0.134	0.211	0.040	0.348*	0.217	1			
12	Playability	0.672**	0.341*	−0.286	0.374*	0.288	0.485**	0.306	0.068	0.492**	0.455**	0.511**	1		
13	Uniqueness	0.421**	0.063	−0.475**	0.570**	0.527**	0.491**	0.595**	0.442**	0.596**	0.564**	0.382*	0.626**	1	
14	Changeability	0.345*	0.194	−0.139	0.463**	0.379*	0.349*	0.342*	0.444**	0.487**	0.437**	0.220	0.372*	0.646**	1

*p < 0.05 (2-tailed), **p < 0.01 (2-tailed).

**Table 4:** Correlation coefficients female participants.

		1	2	3	4	5	6	7	8	9	10	11	12	13	14
1	Place attachment	1													
2	Positive affect	0.566**	1												
3	Negative affect	0.040	0.323**	1											
4	Material	0.505**	0.405**	−0.107	1										
5	Color	0.500**	0.251*	−0.172	0.710**	1									
6	Natural elements	0.522**	0.351**	−0.073	0.741**	0.701**	1								
7	Form and structure	0.606**	0.360**	−0.035	0.723**	0.748**	0.731**	1							
8	Privacy	0.440**	0.266**	−0.126	0.452**	0.349**	0.354**	0.368**	1						
9	Diversity	0.661**	0.489**	−0.068	0.677**	0.548**	0.627**	0.636**	0.495**	1					
10	Sociability	0.584**	0.437**	0.109	0.355**	0.289**	0.433**	0.446**	0.265**	0.557**	1				
11	Territoriality	0.562**	0.330**	−0.177	0.537**	0.398**	0.504**	0.472**	0.455**	0.617**	0.483**	1			
12	Playability	0.644**	0.428**	0.164	0.419**	0.258**	0.294**	0.449**	0.386**	0.595**	0.546**	0.441**	1		
13	Uniqueness	0.620**	0.551**	0.106	0.665**	0.591**	0.692**	0.751**	0.350**	0.692**	0.534**	0.593**	0.578**	1	
14	Changeability	0.441**	0.282**	0.001	0.455**	0.382**	0.409**	0.526**	0.451**	0.522**	0.298**	0.445**	0.471**	0.537**	1

*p < 0.05 (2-tailed), **p < 0.01 (2-tailed).

**Table 5:** Correlation coefficients of participants majoring in environment design related majors.

		1	2	3	4	5	6	7	8	9	10	11	12	13	14
1	Place attachment	1													
2	Positive affect	0.534**	1												
3	Negative affect	0.052	0.246*	1											
4	Material	0.475**	0.255*	−0.084	1										
5	Color	0.500**	0.171	−0.131	0.620**	1									
6	Natural elements	0.466**	0.214	−0.179	0.677**	0.620**	1								
7	Form and structure	0.521**	0.261*	−0.066	0.601**	0.569**	0.692**	1							
8	Privacy	0.246*	0.264*	−0.108	0.344**	0.205	0.273*	0.388**	1						
9	Diversity	0.554**	0.401**	−0.069	0.621**	0.447**	0.526**	0.621**	0.528**	1					
10	Sociability	0.471**	0.365**	−0.110	0.473**	0.442**	0.495**	0.630**	0.346**	0.617**	1				
11	Territoriality	0.484**	0.294**	−0.157	0.525**	0.341**	0.361**	0.334**	0.249*	0.422**	0.320**	1			
12	Playability	0.662**	0.449**	0.062	0.421**	0.358**	0.353**	0.466**	0.254*	0.547**	0.469**	0.368**	1		
13	Uniqueness	0.602**	0.456**	−0.113	0.590**	0.538**	0.628**	0.723**	0.329**	0.663**	0.648**	0.496**	0.657**	1	
14	Changeability	0.280*	0.336**	0.016	0.426**	0.199	0.402**	0.397**	0.368**	0.486**	0.423**	0.349**	0.424**	0.589**	1

*p < 0.05 (2-tailed), **p < 0.01 (2-tailed).

Tables 3, 4 revealed the different mechanisms of emotional attachment to the Heart of Forest among students of different genders. According to the correlation factor data results, the positive emotions of male and female students significantly promoted their emotional attachment to landscape space, while the specific characteristics of landscape promoted the emotions of male students less than that of female students. As for male students, landscape materials and privacy were basically not correlated with their emotional attachment, while color, diversity and changeability were correlated with their emotional attachment but were not as significant as those of female students. Meanwhile, the playability of landscape space in the Heart of Forest have a stronger effect on the emotional attachment of male students than that of female students, and according to the correlation factor between specific landscape features, it can be inferred that the natural elements in the landscape space contribute to the formation of this playful experience. In addition, the color, natural elements and form and structure of the landscape spaces consisting of the Heart of Forest had shown almost no correlation with the sense of territoriality generated by male students, while for female students, the correlation was significant, which also reflected the gender difference of environmental characteristics in promoting the establishment of emotional attachment.

Table 5 described the characteristics and internal mechanism of emotional attachment to the Heart of Forest of students majoring in environment design related majors. Same as the above, these students’ overall attachment to the Heart of Forest was positively correlated with positive affect and most of the landscape characteristics. Among them, unlike the total sample data results, privacy and changeability are the two landscape characteristics that are weaker in correlation with the emotional attachment of students studying environmental design related majors than other characteristics. In addition, surprisingly, the material characteristics of the Heart of Forest such as color, material, natural elements, form and structure were not significantly correlated with the positive emotions of design students, especially color and natural elements hardly promoted their positive emotional experiences here. The correlation calculation results between material and social and interactive characteristics presented by the data of this group of students helped us to further explore how the design and construction method of the Heart of Forest affect these students social and interactive experience. For example, materials, form and structure and landscape diversity showed significant positive correlations, and natural elements, form and structure and landscape uniqueness showed significant positive correlations, indicating to a certain extent that the relevant built environment characteristics that make up the Heart of Forest promoted the design students’ experience of its diversity and uniqueness. While the difference between the total sample data and the design students’ sample data revealed that professional design education may influence students’ emotional experience of the built environment.

#### 4.1.3. The exploratory factor analysis results

Exploratory factor analysis aimed to condense and refine the above spatial factors of landscape characteristics with minimal information loss, and explore the characteristics that play a major role in the emotional attachment process of the landscape characteristics of the Heart of Forest from the bottom up, including both material characteristics and social and interactive characteristics. KMO and Bartlett spherical tests were carried out and the results showed that their structural characteristics were good and met the conditions of factor analysis.

Based on the gravel plot and the rotational component matrix, the main factors (principal components) that played a role in the establishment of emotional attachment to landscape characteristics of the Heart of Forest could be extracted into three (Table 6). The main Factor 1 with the strongest explanatory effect on the establishment of emotional attachment was composed of color, natural elements, form and structure, material, and uniqueness, which mainly describes the built environment characteristics of Heart of Forest’s landscape space and its unique positioning in the campus, indicating that Heart of Forest ‘s landscape space ontology has an important impact on the promotion of users’ emotional experience, and the formation of this emotional experience was closely related to its uniqueness. Among them, color and natural elements had the strongest explanatory effect on this main factor, which was consistent with the characteristics of the landscape space of the Heart of Forest: a variety of plants, water bodies, natural material settings and the use of natural colors. The main Factors 2 that have a relatively strong explanatory effect on the establishment of emotional attachment include playfulness, sociability, territoriality and diversity, which described the interesting interaction characteristics and localization characteristics of the Heart of Forest, and revealed that providing interesting social activity opportunities and creating a place spirit exclusive to campus were conducive to the formation of students’ emotional attachment. The main Factors 3 that had the weakest explanation for the establishment of emotional associations included privacy and changeability. Among them, the effect of privacy on emotional attachment was weak, which was consistent with the results of the aforementioned correlation analysis and the characteristics of the Heart of Forest as an open campus landscape space. However, changeability did not play an effective role in the formation of emotional attachment, indicating that many interactive devices set up in the Heart of Forest did not play an effective role in promoting interactive activities, which might be closely related to its difficult to be detected in spatial distribution and lack of maintenance in subsequent operations.

**Table 6:** Main factors promoting the establishment of emotional attachment to landscape characteristics of the Heart of Forest.

Specific landscape characteristics	Factor 1	Factor 2	Factor 3
Color	0.958		
Natural elements	0.863		
Form and structure	0.769		
Material	0.769		
Uniqueness	0.460		
Playability		0.868	
Sociability		0.824	
Territoriality		0.573	
Diversity		0.512	
Privacy			0.915
Changeability			0.616

### 4.2. The results of sentiment analysis

Using a top-down analysis of the landscape spatial characteristics consisting of the Heart of Forest, combining the above scales to explore the emotional attachment strength of specific landscape characteristics, the relevant texts were located and extracted through words associated with landscape material characteristics, social and interactive characteristics, and design characteristics (Table 7). Then, the text of the reviews was subjected to word segmentation, integration, screening, and assignment calculations to obtain the students’ emotional attention to and emotional experience intensity in different landscape spaces of the Heart of Forest.

**Table 7:** Frequency of representative texts of different landscape characteristics.

Review classification	Related landscape characteristics	Representative text	Frequency
Material	Natural element	Forest	721
		Plant	
		Tree	
		Lawn	
		Birds chirp	
		Insect sound	
		Mosquito	
		Pond	
		Water	
		Swamp	
		Swan lake	
	Time element	Night	362
		Lamplight	
		Spring	
		Autumn	
		Summer	
		Winter	
Social and Interactive	Territoriality	Campus	659
		Memory	
		Old hospital	
		Monuments	
		Campus culture	
	Changeability	Interactive games	458
		Device	
		Sound collection	
		Experience	
		Sensor	
	Sociability	Single	242
		In love	
		Couple	
		Date	
		Party	
		Discuss	
		Delicacies	
	Feeling	Wish	863
		Expression	
		Beauty	
		Nice	
		Emotion	
		Full of life	
		Mysterious	
		Romantic	
Design-related	Design strategy	Landscape	397
		Space	
		Designer	
		Gardens	
		Create	

The frequency of texts helped to understand students’ emotional attention to different landscape characteristics. In general, the high frequency of text associated with students’ feeling and natural element of landscape itself indicated that natural elements in Heart of Forest triggered students’ diverse emotional experiences. At the same time, it could be found that in addition to the material elements in the landscape space, experiences related to animals had also become important emotional triggers. Meanwhile, the low frequency of text associated with social events, especially couple dating, indicated the effect of social activities on students’ attachment to the Heart of Forest is weaker than that of the landscape itself, which also proved the success of the landscape transformation as the environment was highlighted.

**Figure 5:**
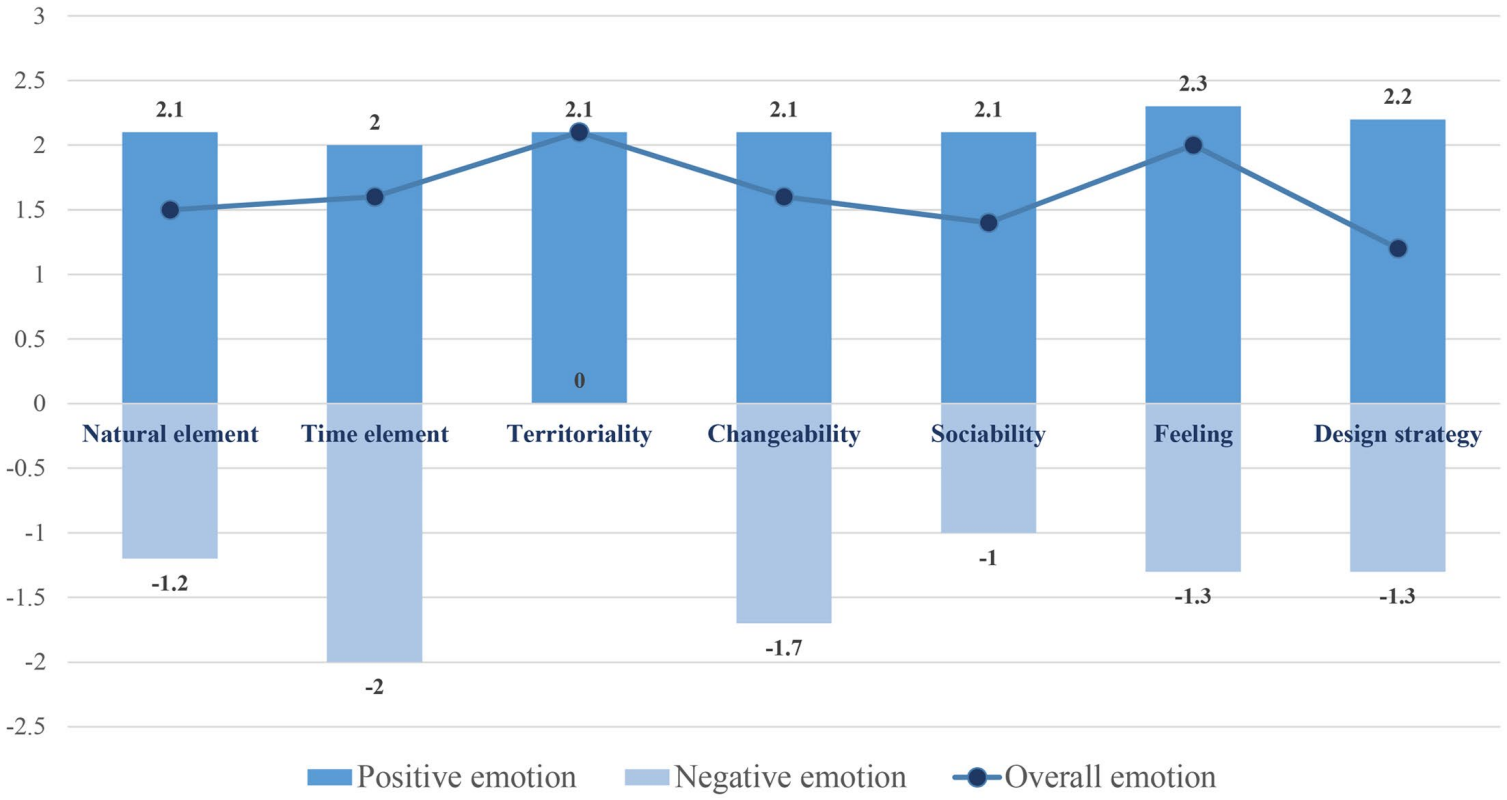
The semantic calculation results.

The sentiment and semantic calculation results for the reviews reflected the positive and negative directions of emotional experiences by the positive and negative numerical values, respectively, and reflected the intensity of emotional experiences by the magnitude of the numerical values (Figure 5). In general, the overall emotional intensity values for the semantic evaluations of the landscape characteristics were all positive, indicating that the emotional experience of students in the Heart of Forest was positive and pleasant. And the “Feeling” had the highest scores, which again proved the positive emotional trigger effect of this landscape space. The students intentionally or unintentionally experienced emotional changes in the process of interactions between individuals and sites and were derived from both the material and social and interactive landscape characteristics. This was consistent with the results of the above scale.

“Feelings” had the highest positive score, and according to its representative texts, it could be inferred that the beauty and romantic atmosphere of landscape characteristics triggered a strong emotional experience during students’ studying in the Heart of Forest. “Design Strategy” also received a relatively high score, indicating that the creation of the landscape had played a positive role in promoting the emotional experience of the students, which complemented a part not mentioned in the previous scale. Meanwhile, the frequent occurrence of the word “designer” in this part once again proved that Professor Cai, who designed the Heart of Forest, had promoted many students’ love for this landscape with his personal charm, which was also consistent with the results of the qualitative interview latter. “Territoriality” had also got a high positive sentiment score and is the only one characteristic without a negative sentiment score. According to its representative texts, the creation of the school’s history, memory and culture-related landscape spaces had become an important emotional anchor point, which once again revealed the importance of the spiritual value of the campus landscape as a “place” to the establishment of students’ emotional attachment. This result also complemented the details not described in the territoriality section of the aforementioned scale. This further confirmed the value of the design concept of “Heart of Forest”: to carry and reproduce campus memories through landscape renewal.

As for negative emotional semantics analysis results, “Time element” and “Changeability” had relatively high negative scores. Combined with observation and in-depth interviews, for the former, season-related mosquitoes and water accumulation caused by heavy rainfall were the main causes of negative emotions. For the latter, the physical and sound interaction facilities originally set up by the designer could not be used normally due to lack of maintenance, which brought a negative experience from the interactive and changeable aspects of the Heart of Forest.

### 4.3. The supplementation and corroboration of interview and observation

The qualitative research method based on observation method and interview method further excavates the emotional triggering characteristics of landscape space that are not revealed by the aforementioned scale data and big data by observing the interaction between students and space at different times in the Heart of Forest and conducting in-depth interviews with students, and at the same time interprets and explains the data results. The qualitative results showed that, overall, the design of the Heart of Forest was relatively successful in terms of promoting emotional attachment. The lawn bathed in sunlight, the pleasant birdsong, the growing trees, and the water surface of the pond were all widely loved by students. Diverse interactive facilities and public spaces for dating and dining had greatly enriched students’ campus life, especially during the COVID-19. In addition, the different landscape styles of the four seasons and day and night of the venue also left users with rich time and space memories.

Qualitative research had also further revealed some problems that site design might trigger negative emotions among users. For example, the most frequently cited problem was the excessive mosquito insect in the summer venue, which created a negative experience for students who wanted a leisure experience. Secondly, although the facilities in the site were abundant, but such as the specimen wall of “Secret Language in the Forest” and some lighting facilities of the site were often damaged, which brought great inconvenience to students. Most students were unfamiliar with the use of interactive facilities such as the “central light column,” resulting in the lack of interactive experience that landscape spaces could provide. In addition, due to the climate of Beijing, the pavilions of the Heart of Forest were less windproof in winter, and the large cold wind and cold climate prevented students from getting a good experience in winter. A small number of students who majored in landscape related majors also reflected the problem of insufficient uniqueness of the landscape, and looked forward to more diversified innovations in design forms. Some students also put forward higher requirements for the balance of landscape places and self-study spaces, as well as insufficient privacy, water accumulation on rainstorm days, and insufficient plant maintenance and cleaning. The interviews had also revealed some new characteristics of the site that promoting students’ positive emotions. For example, we found that purposeful activities could effectively enhance students’ emotional attachment to the landscape space of the Heart of Forest. In addition, the specimen wall in the “Museum in the Forest” and the diverse plant planting in the landscape made the Heart of Forest a new place for teaching practice in the garden and plant related courses, creating a variety of use scenarios for the site.

On the one hand, the results once again support the calculation results of the aforementioned scale and big data, and on the other hand, provide more detailed descriptions of the spatial characteristics of the landscape that promote emotional experience. You may insert up to 5 heading levels into your manuscript as can be seen in “Styles” tab of this template. These formatting styles are meant as a guide, as long as the heading levels are clear, Frontiers style will be applied during typesetting.

## 5. Discussion

The study combines the top-down emotional attachment scale and bottom-up public semantic analysis to collect multi-dimensional emotional interaction data between people and the Heart of Forest to study and evaluate the emotional association characteristics of this landscape space. Observations and interviews are used throughout the data collection and analysis process to complement and interpret quantitative results. The research reveals the factors that promote or weaken students’ emotional attachment to the Heart of Forest from different aspects including material, social and interactive characteristics of the landscape. On the one hand, the result illustrates the positive emotional promotion value of naturalized landscape to college students and the healing effect of nature, especially under the background of COVID-19 epidemic. And on the other hand, it also proves that positive emotional experience is conducive to strengthening students’ emotional attachment to the campus environment, so as to help students obtain stronger place identity. In general, the research results not only support the existing research, but also provide new references for the study of emotional attachment between people and the environment from both the theoretical and methodological level.

At the theoretical level, consistent with previous studies, the healing effect of natural landscapes on negative emotions and the triggering effect of positive emotions have been confirmed again (Kaplan and Kaplan, 1989; Kaplan, 1995). Specifically, nature-related elements, materials, structures and spaces that are easy to get close to nature have important value in providing a good emotional experience. It is achieved by intentionally or unintentionally triggering students to interact with nature, such as watching, touching, listening, etc. (Lee et al., 2009; Koga and Iwasaki, 2013; Honold et al., 2016; Ratcliffe, 2021; Rickard and White, 2021). From the perspective of the material composition of space design and construction, the material characteristics of the site play a positive role in promoting the vast majority of students in the process of establishing emotional attachment (Zhang, 2021; Zhang, 2023). Besides, what’s new of this study is that this promotion effect is particularly obvious for students majoring in landscape, architecture and urban planning, while students in other majors are relatively more interested in the social and interactive characteristics of the Heart of Forest, such as playfulness and changeability as important dimensions for them to gain emotional attachment. It reveals that the emotional experience provided by space is closely related to students’ understanding and cognition. This emotion may be also closely related to the admiration of the designer himself from students studying design-related majors.

Meanwhile, the social characteristics of landscape space in the establishment of emotional attachment is influenced by more subjective and objective factors outside the built environment. This is consistent with the value of the social dimension on attachment establishment in the aforementioned study of place attachment (Scannell and Gifford, 2010; Lewicka, 2011; Lin et al., 2019). For example, whether the public social space created by the designer can effectively provide students with a good emotional experience is closely related to the frequency and purpose of their use of the space (Liu et al., 2020; Arnberger et al., 2022). In addition, for the specific group of college students, multimodal data further illustrate that activities related to teaching and research are not as strong as leisure and entertainment-related activities such as dating and parties to promote place attachment, the negative emotions caused by them are often more obvious, indicating that academic pressure may affect the emotional healing value of landscape space.

In addition, compared with existing research (Birnbaum et al., 2021), this study also reveals the possibility and effectiveness of using new technologies to enhance the emotional attachment between people and the landscape environment in the digital age. For the interactive characteristics such as changeability and playability in the Heart of Forest, the results show that interesting interactive facilities and digital interactive installations originally set up in the design fail to provide students with a positive emotional experience as expected, but become a factor that triggers negative emotions due to the lack of maintenance and complicated use in the later stage. This also shows that before intervening in emerging technologies and new space types in campus landscape space design, it is necessary to conduct more detailed research and consideration of the installation itself and the interactive experience it can provide.

At the methodological level, with the support of big data technology in the digital age (Barbier and Liu, 2011; Chakraborty et al., 2020; Jindal and Aron, 2021), this study updates and improves the research logic dominated by the qualitative dominance of traditional emotional attachment by integrating multimodal data, providing a more scientific, objective and generalizable method (Zhang, 2023). Through the coupling analysis of top-down scale data, bottom-up big data mining and public semantic analysis, and the qualitative results obtained from in-depth interviews and observations, it can be found that overall, the data obtained in different dimensions are mostly consistent, and the different aspects of emotional attachment between people and landscape space are emphasized. The scale data is collected by researchers based on the theory of emotional attachment and purposefully constructed scales according to the research question (Adams, 2015), and its analysis logic is relatively systematic, which is the main research method to explore the mechanism of emotional attachment of this study. The results of qualitative studies are in some ways more in-depth and specific and can provide interpretation and supplement to the scale data. Big data analysis is bottom-up (Wilkins et al., 2020; Zhang, 2021), although the impact of the researcher effect on the data results is avoided, but the content involved in the data is relatively broad, focusing more on the overall evaluation of students’ emotional experience, so it is necessary to combine the description of the spatial characteristics of the landscape in the aforementioned scale to carry out further semantic analysis.

The study improves on the shortcomings in the current post occupancy evaluation of campus renewal spaces especially from the perspective of students’ emotional attachment (Feng et al., 2021; Zhang et al., 2022). The results also provide references for the future design and policy practice. Meanwhile, the study avoids the disadvantages of traditional research methods which are always limited to few data, low efficiency and high costs (Ekkekakis, 2013). It promotes the convergence of bottom-up and top-down quantitative methodological logic while enables large-scale automatic evaluation of landscape spatial quality and public sentiment with the help of sentiment analysis and other technologies. The multi-dimensional analysis and evaluation can reveal the ability of specific landscape characteristics that help promote students’ emotional attachment more completely.

However, there are still limitations to this study which can be promoted in the future. For example, the completeness and rationality of the scale need to be further improved. The landscape characteristics consisting of the emotional attachment scale are not fully representative of the space and may affect the data results. Furthermore, in addition to students, faculty, staffs and their families on campus should also be considered as an important part of landscape users. The number of the scale collected should be expanded after the impact of COVID-19. Also, while the study has tried to screen users who identify as students as data sources, Sina Weibo and Little Red Book (XiaoHongShu) users may also include people other than students, which has an impact on the representativeness and accuracy of public sentiment results in the Heart of Forest. And the choice of social media platform also affects the results of sentiment semantic analysis. Therefore, we will update the emotional attachment scale in the future to include more spatial factors that may influence people’s emotional experience. Meanwhile, we will focus on more diverse population on campus. The collection of SMD will expand to more platforms and more dimensions to extract a more accurate picture of public attitudes toward renovated campus landscape.

## 6. Conclusion

The research characterizes the mechanisms of students’ emotional attachment to different landscape dimensions and spatial characteristics of the “Heart of Forest” through emotional attachment scale and sentiment analysis of text from Sina Weibo and Little Red Book. Based on the correlation and exploratory factor analysis of scale data, semantic analysis of big data and phenomenological analysis of qualitative data, the factors that mainly trigger students’ emotional attachment to the landscape space are revealed. Meanwhile, the landscape characteristics and their values that specifically affect the intensity and dimension of emotional attachment are identified. The coupling analysis of multi-dimensional data further reveals the differences and specific characteristics of different landscape components in emotional attachment for students of different majors and genders. The research results once again prove that natural landscape still has irreplaceable emotional value and healing effect in the information age, and at the same time, it also improves the shortcomings of classical research in dealing with new problems in the digital age at the methodological level. Its main values are manifested in the following three aspects: (1) The real natural environment and materials are still of great importance in the construction and renewal of the public environment of colleges and universities, especially for the mental health and positive emotions of college students. Digitalization has not diminished its existential value, but has highlighted its preciousness. Multimodal data of the study objectively proved that the material characteristics of the landscape space of the Heart of Forest play the most important role in the formation of emotional attachment. Among them, natural materials, colors, structures and biodiversity have the most significant effect. (2) The emotional value of the social characteristics of landscape space in colleges and universities is closely related to whether it can provide students with the activity space that students subjectively demand. When the social activities carried by the space are actively chosen by students, it can often positively promote students’ positive emotional attachment to the space itself. Compared with academic activities, leisure activities have a more positive effect on the establishment of emotional attachment, and the higher the frequency of activities, the deeper the degree of emotional attachment. (3) As for the interactive characteristics of the renewal landscape, whether they can provide students with interesting and gamified space experience as expected by the designer to enhance students’ positive emotional is closely related to the comprehensibility, ease of operation and subsequent maintenance of the interactive devices.

Overall, the renovation of the Heart of Forest has been relatively successful in providing a good emotional experience for students, promoting their emotional attachment to school, and becoming an important emotional healing place for them especially during the COVID-19 epidemic. Due to the specificity of the research subject, the methodological framework and analysis process in this study can be used as a reference for the study of renewal and improvement of landscape environment and public space of other colleges and universities, with policy and practical guidance.

## Data availability statement

The raw data supporting the conclusions of this article will be made available by the authors, without undue reservation.

## Author contributions

RZ responsible for conceptualization, methodology, software, resources, data curation, writing, visualization, and funding acquisition.

## Funding

This work was supported by the National Natural Science Foundation of China under Grant No. 52208005 and Beijing Social Science Foundation under Grant No. 22GLC063.

## Conflict of interest

The author declares that the research was conducted in the absence of any commercial or financial relationships that could be construed as a potential conflict of interest.

## Publisher’s note

All claims expressed in this article are solely those of the authors and do not necessarily represent those of their affiliated organizations, or those of the publisher, the editors and the reviewers. Any product that may be evaluated in this article, or claim that may be made by its manufacturer, is not guaranteed or endorsed by the publisher.

## Footnotes

^1^ https://m.weibo.cn

## References

[ref1] Adams P. C. (2015). Place attachment: advances in theory, methods and applications. Geogr. Rev. 105, 129–132. doi: 10.1111/j.1931-0846.2014.12061.x

[ref2] Altman I. Low S. M. Place attachment: Berlin: Springer Science & Business Media. (2012).

[ref3] Andre E. K. Williams N. Schwartz F. Bullard C. (2017). Benefits of campus outdoor recreation programs: a review of the literature. J. Outdoor Recreat. Educ. Leadersh. 9:7491. doi: 10.18666/JOREL-2017-V9-I1-7491

[ref4] Arnberger A. Budruk M. Schneider I. E. Stanis S. A. W. (2022). Predicting place attachment among walkers in the urban context: the role of dogs, motivations, satisfaction, past experience and setting development. Urban For. Urban Green. 70:127531. doi: 10.1016/j.ufug.2022.127531

[ref5] Barbier G. Liu H. (2011). “Data mining in social media,” in Social network data analytics. United States: Springer, 327–352.

[ref6] Birnbaum L. Wilhelm C. Chilla T. Kröner S. (2021). Place attachment and digitalisation in rural regions. J. Rural. Stud. 87, 189–198. doi: 10.1016/j.jrurstud.2021.09.015, PMID: 32969339

[ref7] Biswas R. Sen S. (2020). Urban eco-psychological attitude during COVID-19 ‘lockdown’: a survey. Int. J. Creat. Res. Thoughts 8

[ref8] Brandisauskiene A. Buksnyte-Marmiene L. Cesnaviciene J. Daugirdiene A. Kemeryte-Ivanauskiene E. Nedzinskaite-Maciuniene R. (2021). Sustainable school environment as a landscape for secondary school students’ engagement in learning. Sustainability 13:11714. doi: 10.3390/su132111714, PMID: 36519450

[ref9] Brindley P. Cameron R. W. Ersoy E. Jorgensen A. Maheswaran R. (2019). Is more always better? Exploring field survey and social media indicators of quality of urban greenspace, in relation to health. Urban For. Urban Green. 39, 45–54. doi: 10.1016/j.ufug.2019.01.015

[ref10] Carmona M. (2019). Place value: place quality and its impact on health, social, economic and environmental outcomes. J. Urban Des. 24, 1–48. doi: 10.1080/13574809.2018.1472523

[ref11] Chakraborty K. Bhattacharyya S. Bag R. (2020). A survey of sentiment analysis from social media data. IEEE Trans. Comput. Soc. Syst. 7, 450–464. doi: 10.1109/TCSS.2019.2956957, PMID: 37577409

[ref12] Ekkekakis P. The measurement of affect, mood, and emotion: A guide for health-behavioral research. Cambridge: Cambridge University Press. (2013).

[ref13] Feng Y. Wang K. Zhang Y. Li X. (2021). An overview of research on rural public space and place attachment: concepts, logics, and associations. Chin. Landsc. Archit. 37, 31–36. doi: 10.19775/j.cla.2021.02.0031

[ref14] Guo W. Ding Y. Yang G. Liu X. (2022). Research on the indicators of sustainable campus renewal and reconstruction in pursuit of continuous historical and regional context. Buildings 12:1508. doi: 10.3390/buildings12101508

[ref15] Guo X. Tu X. Huang G. Fang X. Kong L. Wu J. (2022). Urban greenspace helps ameliorate People's negative sentiments during the COVID-19 pandemic: the case of Beijing. Build. Environ. 223:109449. doi: 10.1016/j.buildenv.2022.109449, PMID: 35937083 PMC9339086

[ref16] Honold J. Lakes T. Beyer R. van der Meer E. (2016). Restoration in urban spaces: nature views from home, greenways, and public parks. Environ. Behav. 48, 796–825. doi: 10.1177/0013916514568556

[ref17] Jiang B. He J. Chen J. Larsen L. Wang H. (2021). Perceived green at speed: a simulated driving experiment raises new questions for attention restoration theory and stress reduction theory. Environ. Behav. 53, 296–335. doi: 10.1177/0013916520947111

[ref18] Jindal K. Aron R. (2021). A systematic study of sentiment analysis for social media data. Materials Today. doi: 10.1016/j.matpr.2021.01.048

[ref19] Kaplan S. (1995). The restorative benefits of nature: toward an integrative framework. J. Environ. Psychol. 15, 169–182. doi: 10.1016/0272-4944(95)90001-2, PMID: 36636425

[ref20] Kaplan R. Kaplan S.. The experience of nature: A psychological perspective. Cambridge: Cambridge University Press (1989).

[ref21] Kaplan R. Kaplan S. Ryan R.. With people in mind: design and management of everyday nature. Washington, DC: Island Press. (1998).

[ref22] Kara B. (2013). Landscape design and cognitive psychology. Procedia Soc. Behav. Sci. 82, 288–291. doi: 10.1016/j.sbspro.2013.06.262, PMID: 37561230

[ref23] Kellert S. R. Wilson E. O.. The biophilia hypothesis. Washington, DC: Island Press (1995).

[ref24] Koga K. Iwasaki Y. (2013). Psychological and physiological effect in humans of touching plant foliage-using the semantic differential method and cerebral activity as indicators. J. Physiol. Anthropol. 32, 1–9. doi: 10.1186/1880-6805-32-7 23587233 PMC3660240

[ref25] Kong L. Liu Z. Pan X. Wang Y. Guo X. Wu J. (2022). How do different types and landscape attributes of urban parks affect Visitors' positive emotions? Landsc. Urban Plan. 226:104482. doi: 10.1016/j.landurbplan.2022.104482

[ref26] Lai K. Y. Webster C. Kumari S. Sarkar C. (2020). The nature of cities and the COVID-19 pandemic. Curr. Opin. Environ. Sustain. 46, 27–31. doi: 10.1016/j.cosust.2020.08.008, PMID: 32874374 PMC7451129

[ref27] Lau S. S. Yang F. (2009). Introducing healing gardens into a compact university campus: design natural space to create healthy and sustainable campuses. Landsc. Res. 34, 55–81. doi: 10.1080/01426390801981720

[ref28] Lee J. Park B.-J. Tsunetsugu Y. Kagawa T. Miyazaki Y. (2009). Restorative effects of viewing real Forest landscapes, based on a comparison with urban landscapes. Scand. J. For. Res. 24, 227–234. doi: 10.1080/02827580902903341

[ref29] Lewicka M. (2011). Place attachment: how far have we come in the last 40 years? J. Environ. Psychol. 31, 207–230. doi: 10.1016/j.jenvp.2010.10.001

[ref30] Lin G. Wu A. Cai K. (2019). Research on place attachment: concept, Progress and trend. Chin. Landsc. Archit. 35, 63–66. doi: 10.19775/j.cla.2019.10.0063

[ref31] Liu S. Ji Y. Li J. Peng Y. Li Z. Lai W.. (2022). Analysis of Students' positive emotions around the green space in the university campus during the COVID-19 pandemic in China. Front. Public Health 10:10. doi: 10.3389/fpubh.2022.888295 PMC9395969 36016888

[ref32] Liu Q. Wu Y. Xiao Y. Fu W. Zhuo Z. van den Bosch C. C. K.. (2020). More meaningful, more restorative? Linking local landscape characteristics and place attachment to restorative perceptions of Urban Park visitors. Landsc. Urban Plan. 197:103763. doi: 10.1016/j.landurbplan.2020.103763

[ref33] McPherson E. G. (1992). Accounting for benefits and costs of urban greenspace. Landsc. Urban Plan. 22, 41–51. doi: 10.1016/0169-2046(92)90006-L, PMID: 28085098

[ref410] Qiangua’s 2022 Xiaohongshu Active User Portrait Trend Report. (2022). Available at: https://www.qian-gua.com/information/detail/1591 (Accessed June 10, 2023).

[ref34] Ratcliffe E. (2021). Sound and soundscape in restorative natural environments: a narrative literature review. Front. Psychol. 12:570563. doi: 10.3389/fpsyg.2021.570563, PMID: 33981262 PMC8107214

[ref35] Rickard S. C. White M. P. (2021). Barefoot walking, nature connectedness and psychological restoration: the importance of stimulating the sense of touch for feeling closer to the natural world. Landsc. Res. 46, 975–991. doi: 10.1080/01426397.2021.1928034

[ref36] Rousseau S. Deschacht N. (2020). Public awareness of nature and the environment during the COVID-19 crisis. Environ. Resour. Econ. 76, 1149–1159. doi: 10.1007/s10640-020-00445-w, PMID: 32836836 PMC7354367

[ref37] Scannell L. Gifford R. (2010). Defining place attachment: a tripartite organizing framework. J. Environ. Psychol. 30, 1–10. doi: 10.1016/j.jenvp.2009.09.006

[ref38] Scannell L. Gifford R. (2013). Personally relevant climate change: the role of place attachment and local versus global message framing in engagement. Environ. Behav. 45, 60–85. doi: 10.1177/0013916511421196

[ref39] Scannell L. Gifford R. (2017). The experienced psychological benefits of place attachment. J. Environ. Psychol. 51, 256–269. doi: 10.1016/j.jenvp.2017.04.001, PMID: 35369239

[ref40] Sim J. Miller P. Swarup S. (2020). Tweeting the high line life: a social media Lens on urban green spaces. Sustainability 12:8895. doi: 10.3390/su12218895

[ref41] Sina Weibo. (2020). Annual Sina Weibo User Report 2020. Available at: https://data.weibo.com/report/reportDetail?id=456 (Accessed June 11, 2023).

[ref42] Tuan Y.-F. (1979). “Space and place: humanistic perspective” in Philosophy in geography. eds. S. Gale and G. Olsson (Berlin: Springer), 387–427.

[ref43] Van den Berg A.. Restorative effects of nature: towards a neurobiological approach. Proceedings of the 9th International Congress of Physiological Anthropology. (2008).

[ref44] Van Leeuwen E. Nijkamp P. de Noronha V. T. (2010). The multifunctional use of urban greenspace. Int. J. Agric. Sustain. 8, 20–25. doi: 10.3763/ijas.2009.0466

[ref45] Vella-Brodrick D. A. Gilowska K. (2022). Effects of nature (greenspace) on cognitive functioning in school children and adolescents: a systematic review. Educ. Psychol. Rev. 34, 1217–1254. doi: 10.1007/s10648-022-09658-5

[ref46] Wilkins E. J. Smith J. W. Keane R. (2020). Social media communication preferences of National Park Visitors. Appl. Environ. Educ. Commun. 19, 4–18. doi: 10.1080/1533015X.2018.1486247

[ref47] Williams D. R. Roggenbuck J. W. Measuring place attachment: Some preliminary results. NRPA Symposium on Leisure Research, San Antonio, TX. (1989).

[ref48] Xie J. Luo S. Furuya K. Sun D. (2020). Urban parks as green buffers during the COVID-19 pandemic. Sustainability 12:6751. doi: 10.3390/su12176751, PMID: 36834254

[ref49] Yan T. Jin H. Jin Y. (2023). The mediating role of emotion in the effects of landscape elements on thermal comfort: a laboratory study. Build. Environ. 233:110130. doi: 10.1016/j.buildenv.2023.110130

[ref50] Ye L. Peng X. Aniche L. Q. Scholten P. H. Ensenado E. M. (2021). Urban renewal as policy innovation in China: from growth stimulation to sustainable development. Public Adm. Dev. 41, 23–33. doi: 10.1002/pad.1903

[ref51] Yi Z. Liu G. Lang W. Shrestha A. Martek I. (2017). Strategic approaches to sustainable urban renewal in developing countries: a case study of Shenzhen, China. Sustainability 9:1460. doi: 10.3390/su9081460

[ref52] You S. Zheng Q. Chen B. Xu Z. Lin Y. Gan M.. (2022). Identifying the spatiotemporal dynamics of Forest ecotourism values with remotely sensed images and social media data: a perspective of public preferences. J. Clean. Prod. 341:130715. doi: 10.1016/j.jclepro.2022.130715

[ref53] Yuanshuo X. (2023). Renewal strategy of learning space in colleges and universities under the digital trend: a case study of old library of North China University of Technology. J. Landsc. Res. 15

[ref54] Zeng X. Zhong Y. Yang L. Wei J. Tang X. (2022). Analysis of Forest landscape preferences and emotional features of Chinese Forest recreationists based on deep learning of geotagged photos. Forests 13:892. doi: 10.3390/f13060892

[ref55] Zhang R. (2021). Research on affective-oriented brutalist architecture:two cases of campus libraries of the University of California. New Archit. 2, 62–67.

[ref56] Zhang R. (2023). Integrating ergonomics data and emotional scale to analyze People's emotional attachment to different landscape features in the Wudaokou Urban Park. Front. Archit. Res. 12, 175–187. doi: 10.1016/j.foar.2022.06.007

[ref57] Zhang R. Dai Y. Sun Z. (2022). Research and evaluation of the mountain settlement space based on the theory of “Flânuer” in the digital age —— taking Yangchan Village in Huangshan City, Anhui Province, as an example. J. Asian Archit. Build. Eng. S1, 1–17. doi: 10.1080/13467581.2023.2217884

[ref58] Zhu X. Gao M. Zhang R. Zhang B. (2021). Quantifying emotional differences in urban green spaces extracted from photos on social networking sites: a study of 34 parks in three cities in northern China. Urban For. Urban Green. 62:127133. doi: 10.1016/j.ufug.2021.127133

